# A FSH-Secreting Pituitary Macroadenoma Causing A Testosterone Deficiency Syndrome

**Published:** 2014-03-09

**Authors:** Xiong Wang, Li Ge, Yuanqing Cui, Cuihong Lang, Cuifang Hao

**Affiliations:** 1 Reproductive Medicine Center in Qingdao University affiliated Yantai Yuhuangding Hospital, Shandong, China; 2 Weifang Maternal and Child Health Hospital, Shandong, China

**Keywords:** Infertility, Pituitary Adenoma, Semen Analysis

## Abstract

FSH-secreting pituitary adenomas can affect sexual and reproductive function. In this article, we have reported the case of a 32-year-old male with secondary infertility. The patient had sexual and reproductive disturbances. The test results of the blood samples indicated obviously decreased testosterone (T) and estradiol (E2) levels. Based on previous hormonal results, the patient received pituitary stimulation and human chorionic gonadotropin (hCG) tests. Both follicle stimulating hormone (FSH) and luteinizing hormone (LH) showed low response during the pituitary stimulation test. The results of the hCG test indicated that T/E2 could recover to a normal level. In addition, this patient was diagnosed with pituitary macroadenoma, which was supported by the pituitary MRI. The man’s sexual and reproductive functions recovered following surgery. The pathological results confirmed that the tumor tissue was an FSH-secreting pituitary adenoma by immunohistochemical staining. The purpose of this report was to review the relative literature and discuss the influence of FSH-secreting pituitary adenomas on hormones through the hypothalamus-pituitary-testis axis.

## Inroduction

The data from WHO shows that male factors account for 50% of infertility cases (1). As an important factor of male endocrine infertility, more attention should be paid to pituitary adenomas. Previous studies have verified that gonadotropin-secreting pituitary tumors are not rare, accounting for approximately 25% of all pituitary tumors (2). In most cases, gonadotropin-secreting pituitary tumors become clinically evident with obvious tumor growth, which results in neurological symptoms and visual field defects (3). In a few cases, the tumor tissues secrete luteinizing hormone (LH) and/or follicle stimulating hormone (FSH) and cause precocious puberty, supra-physiological serum testosterone or large testicles (4). We report the diagnosis and cure of a patient with FSH- secreting pituitary adenoma and testosterone deficiency syndrome that caused his sexual and reproductive dysfunction.

## Case report

A 32-year-old male visited our hospital for infertility for three years following four years of marriage. In the last three years, the patient had lower sexual desire and less intercourse, accompanied by a decreased volume of ejacula- tion, shorter duration of erection (<2 minutes), and weak penis before ejaculation. There were no oppressive symptoms, such as headache, dizziness, and visual disturbances. Physical examinations showed that his beard and pubic hair were thin whereas his Adam’s apple, bilateral testis and breasts were normal. Two seminal reports of the patient before his operation supported the diagnosis of oligoasthenozoospermia (Table 1). The gonadal hormone levels before the operation are shown in table 2.

**Table 1:** Comparison of pre- and post-operation semen analyses


Pertubation	Before operation		3 months after operation		Reference range
	NO.1	NO. 2	NO.1	NO. 2	

**Sexual abstinence (days)**	6	6	6	6	2~7
**Semen volume (ml)**	0.5	0.4	4.0	4.5	≥1.5
**PH**	7.8	8.0	7.5	7.5	≥7.2
**Sperm concentration (10^6^/ml)**	11.1	10.9	36.6	31.3	≥15
**Progressive motility (PR%)**	8	5	42	45	≥32


**Table 2:** Comparison of pre- and post-operation sex hormones


Pertubation	Twice before operation(interval days：7)		After operation	Reference range
	NO.1	NO. 2		

**T（ng/ml）**	0.22	0.24	3.74	2.8~8.0
**E_2_（pg/ml）**	<5.00	<5.00	21.96	7.7~42.5
**PRL（ng/ml）**	17.73	10.01	19.64	4.6~21.4
**FSH（mIU/ml）**	4.06	4.44	3.67	1.5~12.4
**LH（mIU/ml）**	1.88	2.03	2.68	1.7~8.6


The levels of FSH, LH, prolactin (PRL), cortisol, thyroid stimulating hormone (TSH) and growth hormone (GH) were normal. Although the levels of T and E2 were obviously lower than the minimum reference values, they showed significant improvement after the patient received hCG (Table 3). 

**Table 3:** hCG test (5000U im) results


	Before	After

**T (ng/ml)**	0.24	5.93
**E_2_ (pg/ml)**	<5	33.31


**Fig 1:**
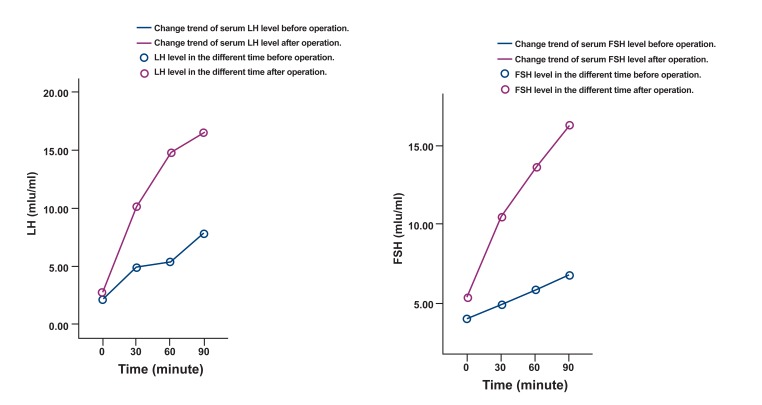
The results of the pituitary stimulation test.

**Fig 2:**
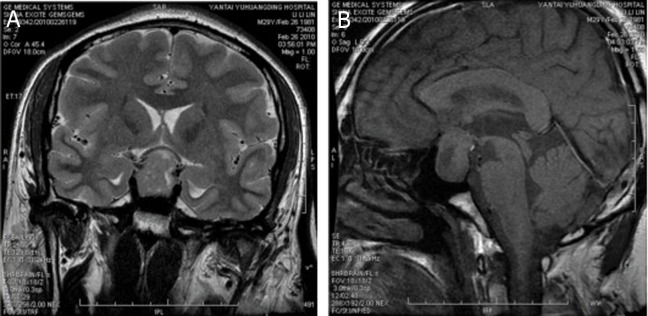
Cornal (A) and sagittal (B) MRI sections revealed ahuge well-enhanced mass in the sella turcica.

The results of the GnRH stimulation test manifested a low response of the serum LH and FSH levels after the administration of 0.1 mg GnRH (Fig 1). The karyotype of this patient was 46 XY. The result of a plain MRI scan revealed that the sella turcica was clearly enlarged, which contained an elliptic lump (2.5 ×2.0 ×1.8 cm) and appeared as slightly longer T1 and T2 signals. In front of the lump, several cycloid cysts appeared as long T1 and T2 signals. The basilar part of the sella turcica clearly sagged and the pituitary stalk was not clearly visible because of space occupation in the sphenoid sinus and sellar area. The optic chiasma was obviously oppressed and its location was moved upward. In addition, the bilateral carotid arteries were encased due to oppression of the surrounding tissues. Based on these signs, a pituitary adenoma was confirmed (Fig 2)

**Fig 3:**
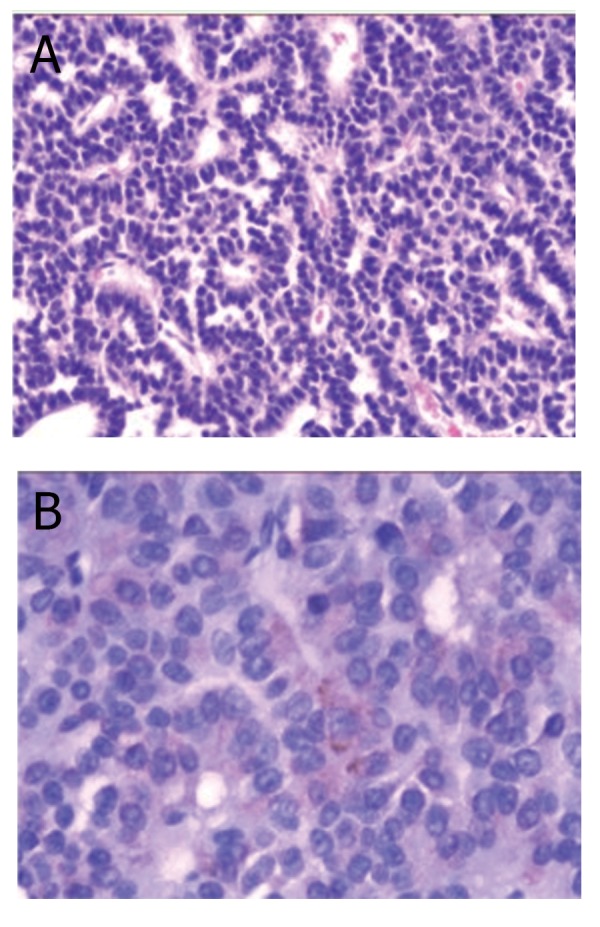
The histologic findings were compatible with pituitary adenoma (A. HE×100). The focus of the tumor cells were positive for β-FSH immunostaining (B. ×400).

The examination relating to the fertility of his spouse, who had been pregnant before their marriage, did not reveal any problems. Thus, the clinical diagnosis was as follows: secondary infertility, erectile dysfunction, oligoasthenospermia and pituitary adenoma. After the initial treatment, he was advised to undergo specific treatment in The Department of Neurosurgery. The neurosurgical removal of his pituitary adenoma was performed by the trans-sphenoidal route with protection of the healthy pituitary tissues. The result of the postoperative pathology was pituitary adenoma (Fig 3A). Immunostaining showed that only β-FSH was positive (Fig 3B) whereas PRL, LH, TSH, GH and ACTH were negative.

**Fig 4:**
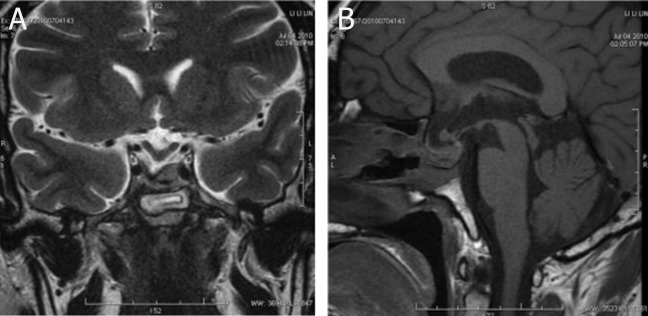
Coronal (A) T2WI showed pituitary gland was shifted to the left side. Sagittal (B) TIWI demonstrated that de- creased signal intensity in sella bottom coincided with post- operative changes

The patient recovered and had a normal sexual life after the operation. His semen improved and became normal three months after the operation (Table 1). The concentrations of FSH, LH, T, E2 and PRL were normal (Table 2). The serum level changes of LH and FSH were normal in the GnRH stimulation test (Fig 1). The results of the followup MR imaging were as follows: the pituitary gland had an irregular shape with bulging in the left side and contained an oval, slightly high single nodule (1.1×0.7 cm); minor sinking of the sella bottom; an obvious right-shift of the stalk and a normal optic chiasma (Fig 4). After treatment, his spouse successfully gave birth to a healthy baby girl naturally.

## Discussion

Pituitary adenomas, as one of the most common intracranial tumors, can be divided into two categories, clinically functional adenomas and nonfunctional adenomas. Functional adenomas mainly include GH-secreting adenomas (GHomas), TSH-secreting adenomas (TSHoams), prolactinsecreting adenomas (PRLomas), ACTH-secreting adenomas (ACTHomas), LH-secreting adenomas (LHomas) and FSH-secreting adenomas (FSHo- mas). Compared with other pituitary adenomas, GHomas and PRLomas are clinically frequent, however the remainder are rare. The rest of the pituitary adenomas are mostly clinically non-functioning. Most clinically non-functioning pituitary adenomas are gonadotrope-derived, while, in most cases, these adenomas secrete low levels of FSH, LH or only the biologically inert alpha- or betasubunits of these hormones. Therefore, most pituitary adenomas are endocrinologically silent and patients commonly present with different symptoms such as impaired vision, headache or hypopituitarism. The diagnosis of a pituitary adenoma is mainly based on the clinical manifestations of the patients, endocrine test results and imaging examinations. It should be particularly emphasized that the majority of clinically non-functional adenomas are confirmed to be positive for gonadotropin subunits by immunohistochemical staining (5).

For the patient in this report, the blood FSH/LH level was within the normal range, and the blood testosterone (T)/estradiol (E2) level was below the lower limit of the reference range. Generally, it was believed that for patients with FSH-secreting pituitary adenomas, the levels of FSH should be increased. However, the FSH level was not elevated in our patient, which could be explained by the increasing degradation of the FSH secreted by the pituitary adenoma cells, which resulted in no change in the FSH level (6). The MRI and immunohistochemical results definitely supported the diagnosis of an FSHoma., The case report of Dahlqvist P showed a typical patient with a large pituitary adenoma combined with signs of hypogonadism, excessive levels of serum FSH and bilaterally enlarged testes. All of the above improved after pituitary surgery (7).

In men, androgen is very important in every phase of life. Testosterone, more than 95% of which is derived from the testes, is by far the most important and abundant androgen in the blood. During the embryonal stage, testosterone determines the differentiation of the sexual organs; during puberty, testosterone furthers the development toward the adult male phenotype, which is then maintained along with the important anabolic functions. Double hydrogen testosterone (DHT) is the main androgen acting on the epididymis, vas deferens, seminal vesicles and prostate, originating from testosterone through 5α-reductase. These tissues are particularly dependent on continuous androgen activity. In the epididymis, seminal vesicles and vas deferens, a lack of testosterone can result in the regression of secretory epithelia, eventually leading to aspermia. The frequency and presence of sexual fantasies, morning erection, frequency of copulation and sexual activity are related to blood testosterone concentrations. Conversely, androgen deficiency is often accompanied by a loss of libido and sexual inactivity. Although axillary hair and the lower part of the pubic hair start growing even in the presence of low androgen concentrations, much higher androgen levels are necessary for the growth of the beard and upper part of the pubic hair.

All of these features were related to the low testosterone levels in the patient’s blood: his beard and pubic hair were thin, and he had persistent hypophrodisia and erectile dysfunction before the operation.

The primary functions of the testis, androgen production and gamete development, are regulated by the brain, e.g., hypothalamus and hypophysis via GnRH and the gonadotropins. Importantly, the hypothalamo-hypophyseal circuit is subject to negative feedback regulation mediated by testicular factors. The site of androgen production in the testis is the Leydig cell. Both its synthesis and secretion are under the regulation of pituitary LH and local factors (8). In men, the main source of estrogens is from the conversion of testosterone into estradiol catalyzed by the enzyme aromatase. T and E2 have independent effects on LH. Whereas the negative feedback by T on LH occurs at the pituitary gland, the negative feedback by E2 on LH occurs at the hypothalamus (9). Given the low serum levels of T and E2 , the serum LH level in the patient in this report should have been higher. Consequently, his hypothalamus-pituitary-testis axis was thought to be abnormal. The LH and hCGβ subunits are structurally very similar, and LH and hCG act on the same receptor. Thus, hCG has a similar biological activity to that of LH. His testosterone and estradiol levels were clearly improved after being injected with hCG, which may suggest that the endogenic LH does not play an effective role in the testicular interstitial cells. Gonadotropin-releasing hormone, which is released by the hypothalamus in pulses through the hypophyseal portal vessel to the adenohypophysis, stimulates gonadotropic cells to secrete LH, which is also released in pulses. According to the results of the GnRH stimulation test before the operation, serum LH and FSH showed low responses after the administration of 0.1 mg GnRH. The concentrations of T/E_2_ were low, and the spermatozoa concentration and motility were poor before the operation; however, these features were normal after surgery, which was related to the normal response of serum LH and FSH in the GnRH stimulation test after surgery. In comparison, we thought that the mechanical compression or non-mechanical factors from adenomas might have affected the pulse secretion of LH, leading to low levels of testosterone and estradiol in the blood before surgery.

In this report, the primary symptom was sexual and reproductive dysfunction without neurological symptoms and visual field defects. Additionally, the FSH level was normal in the blood, but the T level was low. Usui had reported one 40 year-old male with a giant FSH-secreting pituitary adenoma who was admitted to the hospital for vision disorders for two years (10). The hormone tests showed that the FSH level was slightly higher than normal but that the T level was within the reference range, which was inconsistent with the results in our report.

The pituitary FSH-secreting adenomas can be dis- covered in different ages of males. The levels of serum FSH differ significantly and the first diagnosed symptoms can be diverse, such as, headache, dizziness, vision field defect or reproductive dysfunction. The different symptoms of adenomas may be related to the size of the lump and its effect on the normal pituitary tissue and adjacent organs. Further study is needed to determine whether additional factors are involved.

The standard therapy for gonadotropin-secreting macroadenomas (diameter ≥1 cm) is trans-sphenoidal surgery. Because of the generally slow growth of microadenomas (diameter <1 cm), observation accompanied by regular endocrinological monitoring and MRI appears justified in the absence of clinical symptoms. No effective drug therapy for gonadotropin-secreting tumors has yet been established; radiotherapy is only indicated in special cases, such as residual or recurrent tumors after trans-sphenoidal surgery (11). However, pituitary adenomas are an important factor in sexual and reproductive dysfunctions in the male and thus should be paid more attention by doctors in the reproductive medicine field, department of urinary surgery and department of male health. We may obtain a satisfactory curative effect if we provide diagnostic therapy on the basis of identifying the etiological factor. 
